# Analysis of the Machinability of Carbon Fiber Composite Materials in Function of Tool Wear and Cutting Parameters Using the Artificial Neural Network Approach

**DOI:** 10.3390/ma12172747

**Published:** 2019-08-27

**Authors:** Norberto Feito, Ana Muñoz-Sánchez, Antonio Díaz-Álvarez, José Antonio Loya

**Affiliations:** 1 Centre of Research in Mechanical Engineering—CIIM, Department of Mechanical and Materials Engineering, Universitat Politècnica de València, Camino de Vera, s/n, 46022 Valencia, Spain; 2 Department of Mechanical Engineering, University Carlos III of Madrid, Avda. Universidad 30, 28911 Leganés, Madrid, Spain; 3 Department of Continuum Mechanics and Structural Analysis, University Carlos III of Madrid, Avda. Universidad 30, 28911 Leganés, Madrid, Spain

**Keywords:** delamination, tool wear, drilling, machinability, composite, CFRP, neural network

## Abstract

Local delamination is the most undesirable damage associated with drilling carbon fiber reinforced composite materials (CFRPs). This defect reduces the structural integrity of the material, which affects the residual strength of the assembled components. A positive correlation between delamination extension and thrust force during the drilling process is reported in literature. The abrasive effect of the carbon fibers modifies the geometry of the fresh tool, which increases the thrust force and, in consequence, the induced damage in the workpiece. Using a control system based on an artificial neural network (ANN), an analysis of the influence of the tool wear in the thrust force during the drilling of CFRP laminate to reduce the damage is developed. The spindle speed, feed rate, and drill point angle are also included as input parameters of the study. The training and testing of the ANN model are carried out with experimental drilling tests using uncoated carbide helicoidal tools. The data were trained using error-back propagation-training algorithm (EBPTA). The use of the neural network rapidly provides results of the thrust force evolution in function of the tool wear and cutting parameters. The obtained results can be used by the industry as a guide to control the impact of the wear of the tool in the quality of the finished workpiece.

## 1. Introduction

Carbon fiber reinforced polymer composites (CFRPs) are widely used in the industry due to their excellent mechanical properties and good corrosive resistance [1,2]. In particular, they are commonly used in aeronautical and aerospace industries in the manufacturing of aircrafts, ships, space shuttles, and more. However, CFRPs are considered low machinability materials because of the abrasive character of the carbon fibers causing strong tool wear, which changes the geometry of the drill bit and affects the final surface quality [3] of the workpiece. 

Delamination damage is commonly observed during drilling of long fiber reinforced composite materials. This defect affects the structural integrity of the laminate for long-term performance, and results in poor assembly tolerance. Several authors have studied the relationship between delamination and thrust force of the tool during drilling operations. The contributions carried out by Hocheng and Tsao [4,5,6] are especially relevant in which different drill geometries have been analytically modelled and validated with experiments. They established the critical thrust force for several tools at the onset of delamination. Moreover, they also analyzed the effect of chisel edge and a pilot-hole onto the critical thrust force and the resulting damage. Empirical models to describe the correlation between thrust force and input variables (such as spindle speed, feed rate, and parameters of drill bit) have been reported in the literature [7,8,9,10,11], by observing, in all cases, a direct relation between thrust force and delamination. 

The tool wear level also shows an impact on the thrust force. Moreover, cutting edge radius and flank wear of the tool present a strong influence on the delamination and uncut fibers formation (spalling) [12]. The wear mechanism of each tool depends on the different combination among the drill bit material and the workpiece selected, as well as the drilling parameters [13,14,15,16], which considerably complicates the number of cases analyzed. Tsao and Hocheng [17] as well as Chen [18] proved that the flank wear increases the mechanical loads (thrust force and torque). They observed a correlation between the thrust force and the excessive tool wear, as well as its impact on the delaminated area extension. The effect of drill wear on the delamination factor becomes significant at a higher spindle speed. Abrao et al. [14] confirmed that abrasion is the main wear mechanism in CFRP machining, which leads to the increment of the thrust force. Meanwhile Murphy et al. [19] and Fernández-Pérez et al. [20] found that tool coatings did not reduce either tool wear or damage to the composite.

The study of the drilling in CFRP materials is still a challenge of the research community. Since the experimental research in this field is not simple and involves elevated cost and testing time, different methodologies have been proposed in order to improve the obtainment of information in a fast and efficient way. Several methods are commented in the following paragraphs.

In order to minimize the number of tests required, a powerful tool was developed by Taguchi known as the Taguchi experimental design method. This method uses a special design of orthogonal arrays to study the entire parameter space with a small number of experiments. This methodology is used frequently to investigate the drilling of composite materials and the influence of the cutting parameters on delamination [21,22,23]. 

The design of experiments and analysis of variance techniques of Taguchi were used by Rao et al. [24] in a study of delamination. Various drill types, three different feed rates, and spindle speeds were selected, concluding that, for minimizing drilling damage, low feed rates were preferred in the drilling of composite laminates. Davim et al. [7] used the design of experiments approach to select cutting parameters for a free damage drilling process, which reaches a similar conclusion. Enemuof et al. [25] developed a Taguchi’s experimental analysis and multi-objective optimization criteria to obtain a delamination-free drilling in fiber reinforced plastic. 

Sardinas et al. [8] applied genetic algorithms (GA) to model the drilling process of a long fiber reinforced polymer laminate (LFRP). This multi-objective optimization tool showed that the most adequate solution for each particular operation could be selected considering, simultaneously, both the productivity and surface quality. The same methodology was used by Saravanan et al. [26] to optimize the material removal rate as a function of the cutting parameters. 

Feito et al. [27] applied a multi-objective optimization based on the ANOVA design, including the tool wear level to carry out a multi-objective optimization to select optimal ranges of design parameters in order to collectively minimize different output variables. Other authors such as Krishnaraj et al. [28], Krishnamoorthy et al. [29], and Abhishek et al. [30] applied different multi-objective optimization methodologies to establish the influence of different input process parameters on the surface quality in order to find optimum machining conditions.

The Artificial Neural Networks approach is the most used methodology reported in the bibliography [31,32,33,34,35] among the various possibilities. However, not many studies apply this methodology in the machining of composite materials. The ability of artificial neural networks (ANN) to capture complex relationships among input-output data is valuable in manufacturing processes where huge experimental data for process modeling is difficult and expensive to obtain. Moreover, ANN models exhibit up to 40% to 70% improvement in experimental error, as well as nearly 40% improvement in general, which is compared to statistical response surface models.

Some authors integrate Taguchi’s parameter design methods with a back-propagation neural network (BPNN) and Genetic Algorithms to optimize manufacturing processes. Usually this alternative is used to optimize a multiple input-output process parameter design problems. For example, Kuo et al. [36] used this integrated methodology to optimize the dyeing process and predict the quality characteristics of elastic fiber blending fabrics. Chen et al. [37] used the ANN/GA approach in the process parameter settings of plastic injection molding at various length-weight ratios. Ko et al. used this approach [38] to study the process modeling for the growth rate of ZnO thin films in pulsed laser deposition (PLD). However, this methodology requires a huge number of initial data, and takes more time than ANN.

This work implements an artificial neural network for simulating the evolution of the tool wear during drilling carbon fiber-reinforced polymers (CFRP) with a helicoidal drill bit. This methodology is developed for predicting the thrust force through the evolution of the wear (from 0 mm to 0.3 mm), the point angle (between 90° and 140°), and the main cutting parameters. It is shown that the method developed is capable of predicting the thrust force over the entire range of wear from 0 mm to 0.3 mm and the range of point angle between 90° and 140°. This is a significant improvement over previous models where these two parameters are not usually analyzed. The neural network control designed is a support tool for the industry. Visual surface diagrams built with the ANN results can be used to control the impact of the wear evolution, reduce the damage, and avoid the rejection of the component.

## 2. Experimental Details 

**Figure 1:**
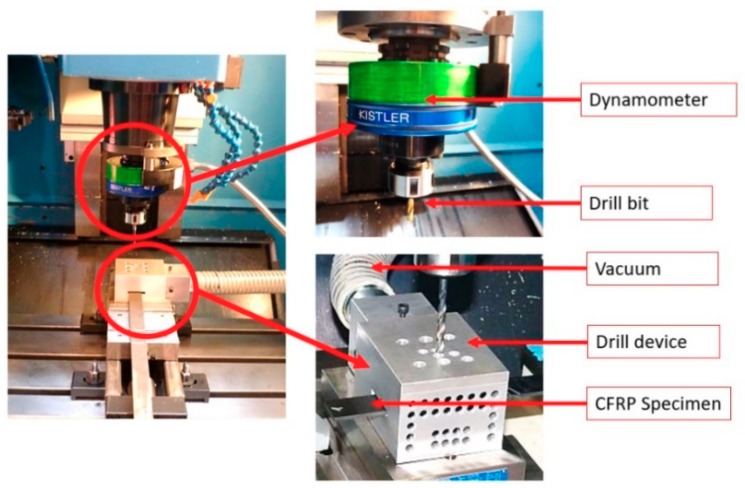
Experimental set-up device.

The process begins by obtaining an input-output database required for training and testing the ANN. For this study, drilling tests were conducted on a B500 KONDIA machining center (Kondia, Elgoibar, Spain) equipped with a rotating dynamometer Kistler 9123C (Kistler Group, Winterthur, Switzerland) to measure both the thrust force and torque on the rotating tool (Figure 1).

The used material is made of 10 plies (0°/90°) of AS-4 carbon fibers with 55.29% weight and 8852 epoxy matrix. The specimen dimensions are 120 mm × 29 mm and 2.2 mm thickness. The main mechanical properties of the CFRP workpiece are presented in Table 1.

Uncoated carbide twist drill bits with a nominal diameter of 6 mm and 30° helix angle, manufactured by GUHRING, were used as cutting tools. All tests have been performed in a dry environment without using cutting fluids. Three different values of the point angle and wear stage evolution were tested at a different feed rate and cutting speed conditions, which leads to 81 different combinations that will be used to train and check the neural network. Levels 1 to 3 were assigned for these parameters, which are summarized in Table 2. The wear levels were determined from experimental works [15,39] and the cutting conditions were established following the recommendations of the drill’s manufacturer GUHRING for drilling CFRPs and related studies [7,14]. 

## 3. Design of the Artificial Neural Network

A neural network is a technique inspired by the biological nerve system, which intend to replicate the way humans learn to solve a wide variety of complex scientific problems. 

**Figure 2:**
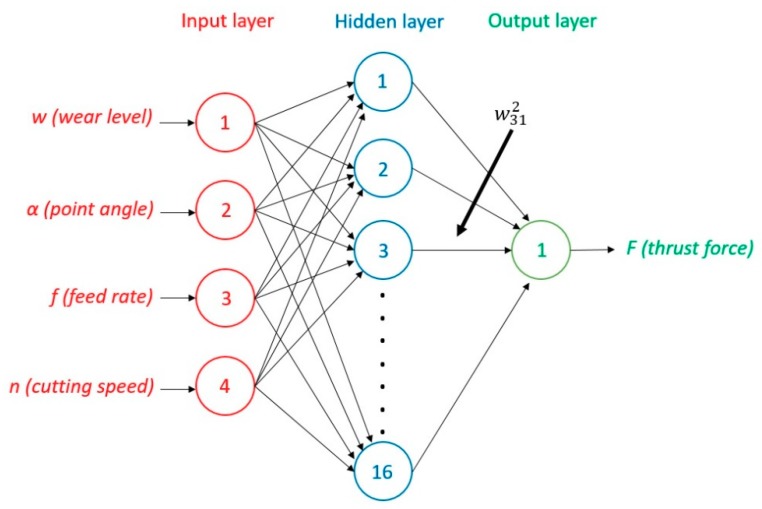
Architecture of the ANN with three layers implemented.

Neural networks consist of several layers of neurons connected to each other with synaptic weights to simulate the human brain. A simplified network consists of an input layer with a number of neurons according to the input variables (4 in our case), which is followed by one or several hidden layers that transform those variables for their final use in the output layer. Figure 2 shows the ANN scheme used, where *w*^2^_31_ is the weight from the third neuron in the second layer to the first neuron in the third layer.

Using the general notation, the activation *a^l^_j_* of the *j^th^* neuron in the *l^th^* layer is related to the activations in the (*l* − 1)^*th*^ layer following Equation (1). $f\left(x_{j}^{l}\right)$ is the activation function applied over $x_{j}^{l}$ neuron, and must be selected by the designer of the network. In this formula, *b^l^_j_* represents the bias of the *j^th^* neuron in the *l^th^* layer, *n*^*l* − 1^ is the number of neurons in the (*l* − 1)^*th*^ layer, $w_{ij}^{l-1}$ are the weights that join the *i^th^* neuron of the (*l* − 1)^*th*^ layer and the *j^th^* neuron of the *l^th^* layer, and $a_{i}^{l-1}$ is the activation of the *i^th^* neuron of the (*l* − 1)^*th*^ layer.

(1) $$\begin{array}{c} a_{j}^{l}=f\left(x_{j}^{l}\right)\\ x_{j}^{l}=b_{j}^{l}+\overset{n^{l-1}}{\underset{i=1}{\sum }}w_{ij}^{l-1}\cdot a_{i}^{l-1} \end{array}\}\to a_{j}^{l}=f\left(b_{j}^{l}+\overset{n^{l-1}}{\underset{i=1}{\sum }}w_{ij}^{l-1}\cdot a_{i}^{l-1}\right)$$

The multi-layer feed forward ANN architecture used in this study consists of four neurons in the input layer, according to the number of input variables (wear level, point angle, feed rate, and cutting speed), which includes one neuron in the output layer (thrust force) and one hidden layer with 16 neurons. It has been found in previous research that the use of one hidden layer provides very good results and simplifies the complexity of the neural network [33,40]. The selected number of neurons of the hidden layer is determined by a trial and error procedure. In this study, after trying different numbers of neurons in the hidden layer, 16 neurons presented satisfactory training. It has been reported that too few neurons can lead to under-fitting the results whereas too many neurons can contribute to over-fitting them. The highly sensitive of the ANN to the number of neurons used in the hidden layer is a relevant property to consider in the design of the network [33].

In order to relieve the training difficulty and balance the importance of each parameter during the training process, the experimental database was normalized between values 0 and 1. The equation to scale input and output variables in the interval is shown in Equation (2), where *V_norm_* is the normalized value, *V* is the real value, and *V_min_* and *V_max_* are the minimum and maximum values, respectively, for the full range of experiments.

(2) $$V_{norm}=\left(V-V_{min}\right)/\left(V_{max}-V_{min}\right)$$

The output of each neuron of the hidden and output layers is given by the logistic sigmoid function, which is defined in Equation (3). Therefore, in this study, the sigmoid function is the activation function detailed below.

(3) $$a_{j}^{l}=f\left(x_{j}^{l}\right)=1/\left(1+e^{-x_{j}^{l}}\right)$$

Following the Levenberg-Marquardt optimization process [41], the error-back propagation training algorithm (EBPTA) was used to train the multi-layer neural network to calculate the needed gradient for calculating the weights.

### 3.1. ANN Training

The ANN training phase primarily determines the connection weights that are required to give the desired response. The first step assigns random weight values to all the links between neurons. Then, the values of the parameters from the *k^th^* experiment in the training data list (w, α, f, and n from Figure 2) are passed through the network. The estimated value is compared with the desired value using the error function, δ, (Equation (4)) where $a_{1}^{3}$ is the activation of neuron 1 of the output layer (no.3) and $o_{1}$ is the desired output for the *k^th^* experiment.

(4) $$\delta =0.5\cdot {\left(o_{1}-a_{1}^{3}\right)}^{2}$$

This error is associated with the neuron of the last layer of the neural network and is distributed to the elements in the hidden layer using a back propagation technique. The error for neuron $x_{i}^{l}$ can be calculated using the equation below.

(5) $$\delta_{i}^{l}=f^{\prime }\left(x_{j}^{l+1}\right)\cdot w_{ij}^{l}\cdot \delta_{j}^{l+1}$$

where $\delta_{j}^{l+1}$ is the error associated with the $x_{j}^{l+1}$ neuron. $w_{ij}^{l}$ is the weight associated with the line from neuron $x_{i}^{l}$ to neuron $x_{j}^{l+1}$. is the sigmoid function, and $f^{\prime }\left(x_{j}^{l+1}\right)=f\left(x_{j}^{l+1}\right)\cdot \left(1-f\left(x_{j}^{l+1}\right)\right).$

The different weights $w_{ij}^{l}$ connecting different elements in the neural network are corrected and got more closely to the target output value. To update the weights, the error computed on each neuron is used, following Equation (6), where $a_{i}^{l}$ is the *i^th^* neuron in the *l^th^* layer, *η* is the learning rate, and $\delta_{j}^{l+1}$ is the error term from the (*l* + 1)^*th*^ layer.

(6) $${\left(w_{ij}^{l}\right)}_{updated}=w_{ij}^{l}+\eta \cdot a_{i}^{l}\cdot \delta_{j}^{l+1}$$

**Figure 3:**
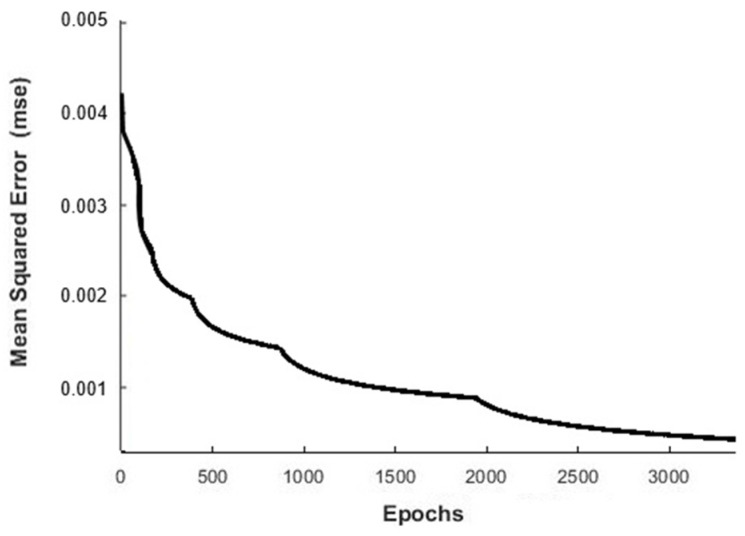
Mean square error evolution with the number of epochs.

After all the weights are updated with each training test using Equation (6), an epoch, *p*, is completed. An epoch is when all training tests (70 for this study) are evaluated. The mean square error (*MSE_p_*) of the network is then calculated as:

(7) $$MSE_{p}=\frac{1}{70}\overset{70}{\underset{k=1}{\sum }}{\left(o_{k}-a_{k}^{3}\right)}^{2}$$

where $o_{k}$ is the desired output of the *k^th^* test and $a_{k}^{3}$ is the activation of the last layer neuron for the *k^th^* test. If the MSE is not more minor than a specific goal, the process is repeated updating the weights and increasing the number of epochs until the objective is achieved. The evolution of the MSE with the epochs for the ANN designed is presented in Figure 3, where the convergence of results is observed.

It is important to limit the number of epochs because a prolonged training could result in an ANN that tends to memorize the input-output pattern (overlearning), which loses prediction ability. In the present study, the limit was stablished in 10,000 epochs. A resume of the main factors of the ANN designed can be seen in Table 3. These factors are similar to those that can be found in the literature [33,35].

**Figure 4:**
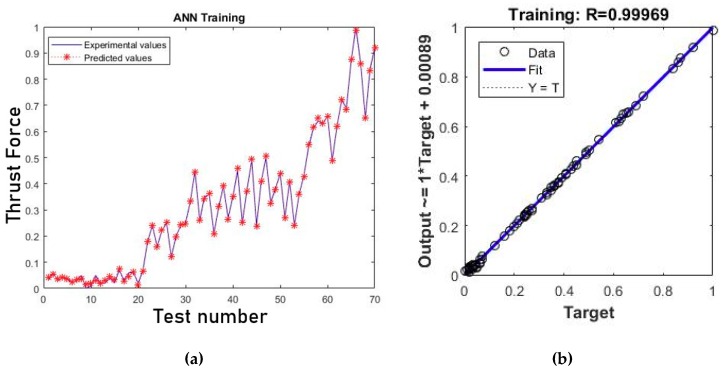
(**a**) Comparison between predicted and experimental values of normalized thrust force for the training pattern and (**b**) correlation analysis of the results.

Figure 4a shows the results for the training process when the minimum gradient was reached. The MSE at the end of the training procedure was found to be 0.423 × 10^−4^. To measure the accurary of the ANN, the correlation coefficient (R-value) between the outputs and targets has been calculated. In the present case, R-value = 0.99969 represents almost a perfect correlation between experimental and estimated values, as can be seen in Figure 4b.

### 3.2. ANN Testing

**Figure 5:**
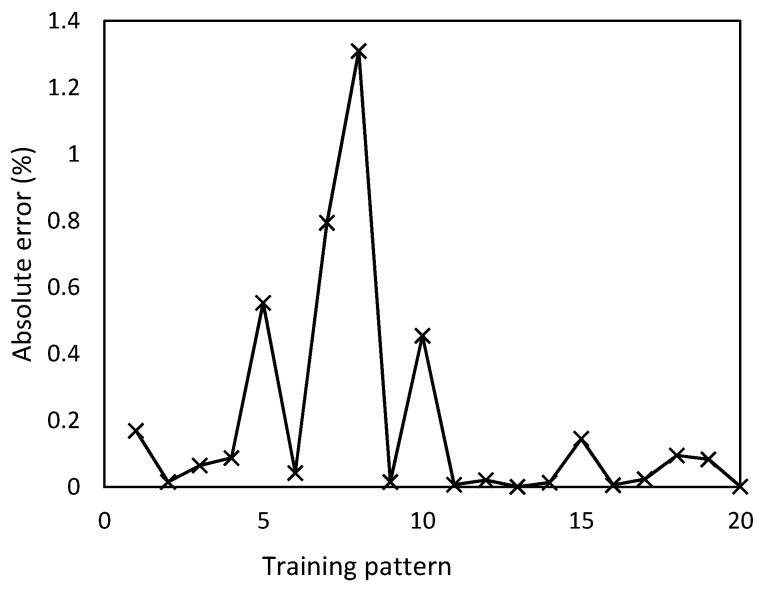
Absolute error profile of thrust force for different training patterns.

The ANN testing phase is divided in two parts. First, the trained ANN was tested with 20 input patterns, which were employed for a training purpose. For each input pattern, the predicted value of thrust force is compared with the respective experimental value. The absolute percentage error was determined by Equation (8) where *F_exp_* is the experimental value of thrust force, and *F_pred_* is the predicted value of thrust force by the ANN model. It can be observed in Figure 5 that predicted values of thrust force are very close to experimental values, where the maximum error committed is 1.2%.

(8) $$\%Absolute\;error=\left|\left(F_{exp}-F_{pred}\right)/F_{exp}\right|\cdot 100$$

**Figure 6:**
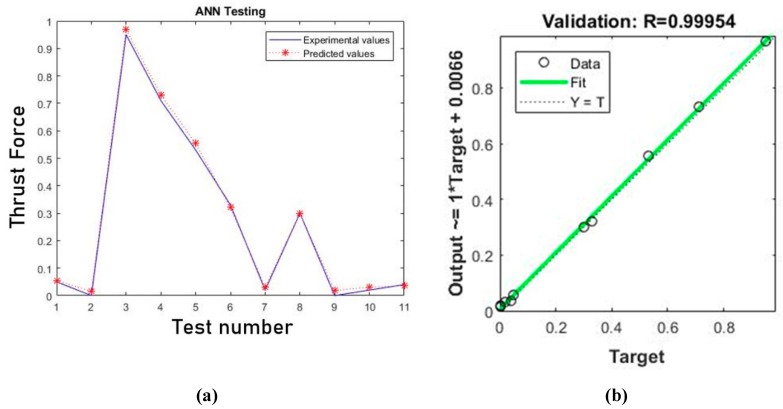
(**a**) Predicted and experimental values of normalized thrust force for the testing pattern and (**b**) regression analysis of the testing results.

Second, the trained ANN was tested using 11 trials that were not adopted for the training phase to validate the artificial neural network. Figure 6a presents the comparison of the predicted and the experimental values of thrust forced, whereas Figure 6b shows the regression analysis for the testing case. It is clearly observed that the predicted values are very close to experimental values supported by the R-value of 0.99954 in the regression analysis. Results presented in both graphics confirm the validation of the model.

## 4. Results and Discussion

**Figure 7:**
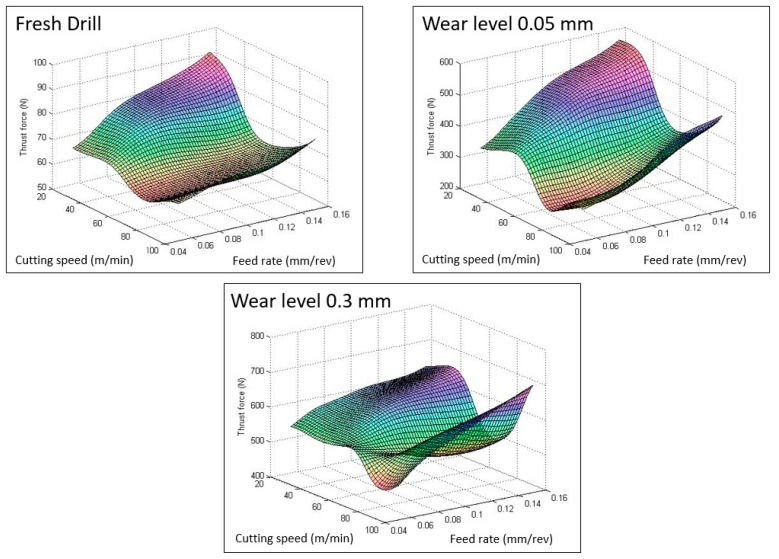
Variation of thrust force with cutting parameters for different wear levels (point angle = 118°).

The artificial neural network designed has been used to study the interaction effect among different parameters in the thrust force. Figure 7 shows the surface diagram of the thrust force estimated by ANN as a function of the cutting variables for the same wear levels fixed in the experiments. In concordance with literature, it can be seen that, usually, the feed rate has a direct effect on thrust force independent of the wear level [3]. The increment of the force becomes pronounced at low cutting speeds. This behavior was observed by Karnik et al. [33] at high speed drilling in CFRP laminates and can be explained by the fact that increasing speed leads to higher temperatures, which promotes the softening of the matrix and, thus, diminishes the force and the delamination. It is also observed, for the three wear levels, that, at high feed rates and high cutting speed, the force increases again. This is mainly due to the built-up edge formed on the tool due to the heat generation, which increases the thrust force [33].

On the other hand, the wear level presents a strong effect in thrust force. The effect of the drill bit pre-wear on the thrust force during drilling GFRP materials at different cutting speed, under a constant feed rate, was studied by Khashaba et al. [42]. In this study, the thrust force increased noticeably with greater cutting speed when using pre-wear drill bits. In Figure 7, the results present the same behavior not only when the spindle speed is greater but also when the feed rate is increased.

**Figure 8:**
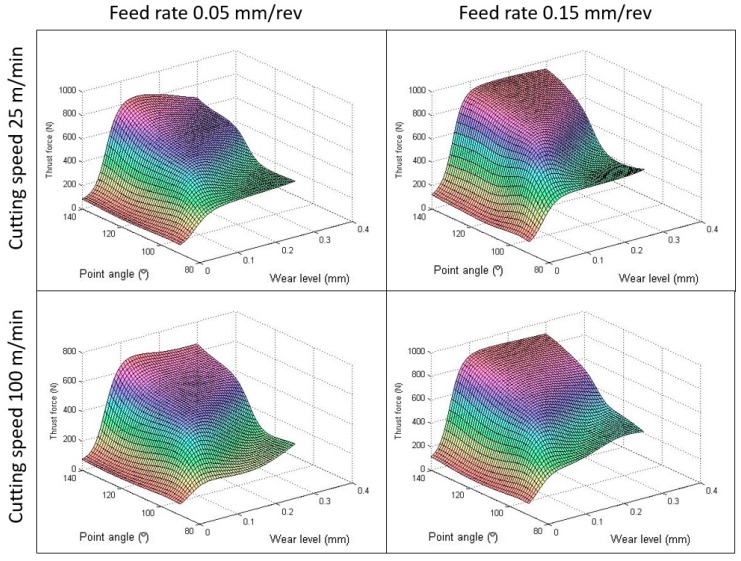
Interaction effects of the point angle and wear level on thrust force.

The cross effect of the tool wear was studied with other parameters in depth. Different studies were taken by varying one parameter, and, at the same time, keeping the rest of the variables constant. It can be observed in Figure 8 that the tendency of the thrust force is to increase with increments on the point angle independently of the tool wear level. However, the point angle influences the wear rate. For small point angles, the increase of the thrust force with the wear is smaller than those cases with higher point angles. This is due to the axial force in tools with high point angles being higher than tools with small angles, where the forces are also axial deflected [43]. In this figure, it is also possible to observe the negligible influence of the point angle on thrust force when the fresh drill is used, but it is relevant with the wear. In addition, the effect of the drill point angle was more significant when the feed was incremented than when the cutting speed was increased [44].

**Figure 9:**
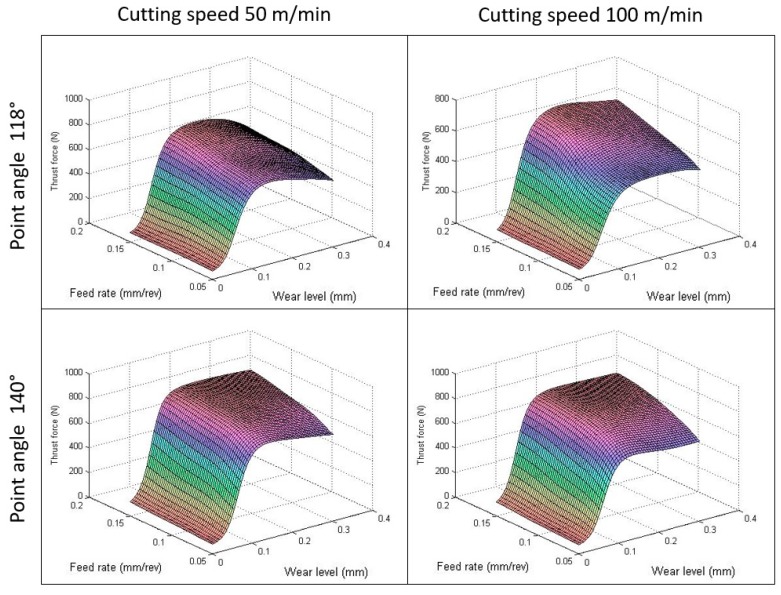
Interaction effects of the feed rate and wear level on the thrust force.

**Figure 10:**
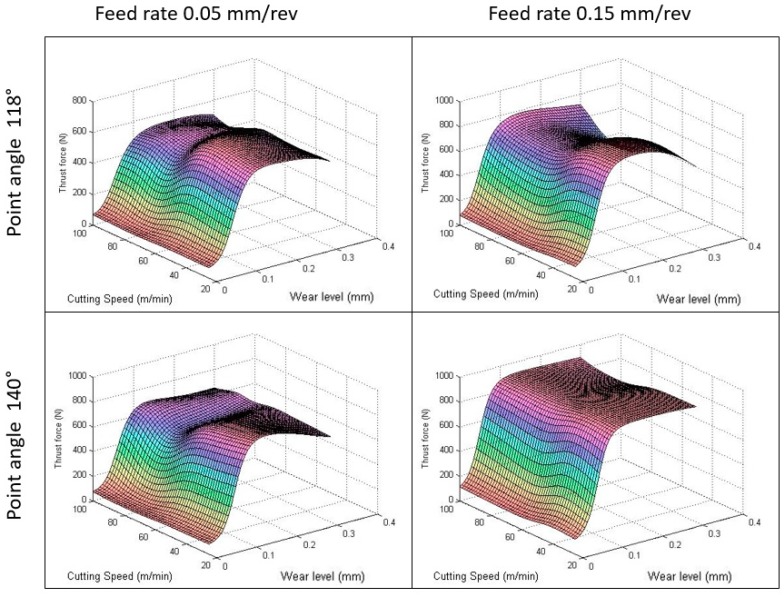
Interaction effects of cutting speed and wear level on the thrust force.

Lastly, the cross effect between the cutting parameters and the tool wear are analyzed. For both cases, the thrust force follows almost a similar pattern as can be observed in Figure 9 and Figure 10. The axial force is highly sensitive to tool wear, which increases quickly in the “primary wear region”. In the first stage, the tool presents a sharp corner radius that involves a small chip contact area resulting in low cutting forces and a high wear rate [15]. 

In the second wear stage (the steady wear region), the force remains almost constant and grows with the feed rate as well as, to a lesser extent, with cutting speed. In this stage, the contact area between the tool and workpiece increases, which results in lower contact stresses and a reduced wear rate becoming nearly constant over time [15].

As mentioned, the influence of the cutting parameters is much more relevant in worn tools, where changes in the feed rate or cutting speed generates changes up to 300 N in some cases. While an increase in the feed rate involves a direct effect in thrust force, selecting middle values of cutting speed can generate a slight reduction in thrust force. Therefore, independently of the initial parameters used, when the wear progresses in the tool, it is recommended to reduce the cutting speed around 60 m/min to minimize the thrust force. The workpiece bending will be reduced, and the damage will be decreased.

## 5. Conclusions

This study presents the design of an artificial neural network model to predict the thrust force when drilling woven CFRP materials with worn tools. The robustness of the network was demonstrated by varying the drilling parameters. The following conclusions were determined.

1. As a general recommendation, a low point-angle seems to be an appropriate selection to avoid excessive damage in the CFRP laminate.
2. The effect of the drill point-angle loses relevance with the inevitable evolution of tool wear. This effect demonstrated the significance of the general drill geometry.
3. When tool wear increases, the parametrical analysis of the influence of cutting parameters revealed that the thrust force is more sensitive to feed rate than to cutting speed
4. Therefore, the wear level evolution is the most significant input factor of thrust force, due to the change generated in the tool geometry. However, a critical wear value exists for which the feed rate becomes more relevant than the cutting speed, and the thrust force remains almost constant.
5. The feed rate is the cutting parameter that should be modified when the drill bit wear increases.

The artificial neural network could be a useful tool in machining optimization. By defining more outputs, (i.e., delamination or torque) and including more input variables related to the geometry of the drill bit (i.e., chisel edge or helix angle), it is possible to get a complex prediction model. However, this will need more experiments to validate the model and will increase the cost and time of conducting the experiments.

## Figures and Tables

**Table 1:** Mechanical properties of the CFRP laminate.

Parameter	Definition	Values
E_11,22_	Young’s modulus	68 GPa
G_12_	Shear modulus	5 GPa
υ_12,21_	Poisson ratio	0.05
X_t_ = Y_t_	Maximum tensile strength	795 MPa
X_c_ = Y_c_	Maximum compressive strength	860 MPa

**Table 2:** Mechanical parameters of the CFRP laminate.

Parameter	Level		
	1	2	3
Wear (mm)	0	0.05	0.3
Point angle (°)	90	118	140
Feed rate (mm/rev)	0.05	0.10	0.15
Cutting speed (m/min)	25	50	100

**Table 3:** Main factors of ANN being implemented.

Factor	Value
Learning rate	0.001
Learning rate increment	10
MSE goal	10^−5^
Maximum number of epochs	10,000
